# A Descriptive Comparison of Mass Testing During the COVID-19 Pandemic in Montreal, Paris, Bamako, and Recife

**DOI:** 10.3389/ijph.2022.1604992

**Published:** 2022-09-23

**Authors:** Ashley Savard Lamothe, Morgane Gabet, Zoé Richard, Sydia Rosana de Araujo Oliveira, Abdouramane Coulibaly, Gisèle Cazarin, Amanda Zacarias, Lara Gautier, Valéry Ridde, Kate Zinszer

**Affiliations:** ^1^ Department of Social and Preventive Medicine, School of Public Health, University of Montreal, Montreal, QC, Canada; ^2^ Institut de Recherche pour le Développement (IRD) Université de Paris, Paris, France; ^3^ Centre Population et Développement (Ceped), Paris, France; ^4^ Institut Aggeu Magalhães, Oswaldo Cruz Fondacion, Recife, Brazil; ^5^ Miseli Research NGO, Bamako, Mali; ^6^ Centre de Recherche en Santé Publique (CReSP), Montréal, QC, Canada

**Keywords:** public health, COVID-19, comparative analysis, mass testing, TIDiER-PHP

## Abstract

**Objective:** The aim of this descriptive article was to compare mass testing for SARS-CoV-2 during the first wave of the COVID-19 pandemic in Montreal, Canada; Bamako, Mali; Paris, France; and Recife, Brazil.

**Methods:** Data was collected through interviews with key informants involved in the testing response and a review of the grey literature. The TIDieR-PHP checklist was then used to provide the basis of the intervention descriptions and to compare the data between cities.

**Results:** Descriptive comparisons revealed that the type of test, the testing process, and materials used were similar between the cities during the first wave of the pandemic. In addition, all cities experienced similar material and personnel resource shortages, directly affecting testing accessibility and capacity. The main differences were related to testing capacity and implementation timelines, which were dependent on the state of the health care systems, governance, and access to resources.

**Conclusion:** Results of this study highlight the similarities and differences in testing between the cities and demonstrate the importance of comprehensive intervention descriptions to highlight lessons learned, increase knowledge sharing, and inform policy decisions.

## Introduction

From the beginning of the COVID-19 pandemic, mass SARS-CoV-2 testing has been instrumental in the early isolation and treatment of those who are infected, in informing control measures, and in understanding viral transmission [1]. However, this scale of testing requires a massive mobilization of material, as well as organizational and human resources. Although studies have compared the pandemic response across countries, there are few that have documented public health interventions in a descriptive manner or have used a standard documentation protocol. Studies have compared response strategies, government policies, and the response of citizens to government policies across countries [2–4]. In terms of testing strategies, there have been studies that compared testing rates across countries as well as testing coverage [5], however, most are quantitative in nature and do not compare the key features of the testing response efforts. This paper provides a descriptive comparison of key features of SARS-CoV-2 reverse transcription-polymerase chain reaction (RT-PCR) testing (the most frequently used test during the first wave due to its high sensitivity) strategies during the first wave of the pandemic from four different cities in four different countries: Montreal, Canada; Paris, France; Recife, Brazil; and Bamako, Mali. These countries were chosen to represent different contexts, epidemiological situations (see Table 1), and continents, and these major cities were chosen to facilitate data collection by members from the international research team present in each of the four cities.

**TABLE 1:** COVID-19 context in each city (Montreal, Bamako, Paris, Recife, 2020) [33–36].

	Montreal	Bamako	Paris	Recife
Total population of city (2020)	1,825,208	2,713,000	2,185,574	1,653,461
First reported case of COVID-19 in the city	27th February 2020	25th March 2020	24th January 2020	12th March 2020
Cumulative number of confirmed cases per 100,000 people by 22nd August 2020	1,623	49	Data not found for this time period	1,846
Cumulative number of confirmed deaths per 100,000 people by 22nd August 2020	191	2	82	135
Total number of tests performed by 22nd August 2020 per 100,000 people	63,077	476	Data not found for this time period	Data not found for this time period

## Methods

To draw comparisons on the SARS-CoV-2 testing programs implemented in different countries and contexts, a standardized and thorough description was needed to identify key differences and similarities. There are multiple well-known guidelines for reporting on interventions. Some of the most well-known ones include CONSORT [6], SPIRIT [7] and TIDiER [8]. The Template for Intervention Description and Replication for population health and policy interventions (TIDieR-PHP) framework was created as an adaptation to the original TIDieR guideline to specifically report on public health interventions [9, 10]. This tool is a 9-item checklist that was developed to ensure that key features of an intervention’s design and implementation are being reported on in a consistent manner that will allow findings to be generalized to other contexts. The checklist includes the following categories: brief name, why (rationale), what (materials), what and how (procedure), who provided, where, when, adaptations, and how well.

This study used the TIDieR-PHP checklist and focused on the early stages of the pandemic (i.e., March 2020–22 August 2020) to document the initial response of countries to the unprecedented health emergency. Dates of the first wave vary between the four countries as they each had different epidemiological situations. In Canada and France, the end of the first wave was in mid-August, in Mali in September, and in Brazil in late October. Therefore, to facilitate comparisons the Montreal cut-off date of 22 August 2020 was chosen to mark the end of the first wave. Descriptions were based on a review of grey literature (i.e., official government databases and documents) and on 30–45-min exploratory semi-structured interviews (i.e., with no pre-determined questions). The interviews were conducted by members of the research team with 3–4 informants (i.e., public health professionals involved in SARS-CoV-2 testing programs) in each of the cities. Purposeful sampling was used to recruit the informants. The TIDeR-PHP was then used to analyze data from both data sources and produce the intervention descriptions for each city. Crude rates were calculated by dividing the indicator (i.e., confirmed cases, deaths, and tests performed) by the total number of people in the population multiplied by 100,000.

## Results

### General Context

During the first wave of the pandemic, a shortage of material resources required for SARS-CoV-2 testing (e.g., testing facilities, swabs for oropharyngeal and nasopharyngeal samples, transportation materials, laboratories and laboratory equipment, analysis kits, etc.), personal protective equipment, and human resources were observed across the four cities. This directly affected testing priorities, wait times to get tested and receive results, and laboratory analysis, which in turn impacted the accuracy of confirmed case numbers, the spread of the virus and public health measures [11–14].

### Montreal, Canada

#### What Materials

Information materials used for mass SARS-CoV-2 testing in Montreal included a variety of documents and government resources for the general population in terms of locating a testing clinic, auto-assessing symptoms, and measures to follow while waiting for the test result [15]. These resources were available to the public online or in a paper format at health centers. These materials were made available to the public in up to 18 different languages from the beginning of the pandemic.

#### How It Was Planned

In Montreal, testing priorities constantly evolved according to the epidemiological situation and testing capacity. In March 2020, testing priorities targeted symptomatic hospital patients, healthcare professionals, long-term care facility residents, and travelers and their symptomatic contacts. Soon afterwards, as community transmission increased, travelers were no longer a priority and testing was reserved for health care professionals who had been in direct contact with positive cases. In May 2020, all symptomatic individuals could get tested and asymptomatic individuals who had been in contact with an infected individual could also be tested.

The testing process involved the following steps: 1) identifying individuals who needed to be tested; 2) determining their eligibility; 3) performing the oropharyngeal and nasopharyngeal swabs; 4) identifying and managing samples; 5) communicating test results; 6) and providing psychosocial support as needed [16]. For a negative result, the information was communicated by email or telephone. For a positive result, the information was communicated by telephone only. Online social and community services resources as well as free and confidential telephone consultations were available to those who needed additional support or advice.

#### Who Implemented

In Canada, health care falls under provincial jurisdiction rather than federal, meaning that the health and social services including testing for SARS-CoV-2, are delivered by provinces and territories. In Quebec, the Ministry of Health and Social Services (MSSS) who is at the head of all health-related decisions in the province, oversaw the deployment of testing clinics at the provincial level. In Montreal, there are five Integrated University Health and Social Services Centres (CIUSSS) that coordinated testing clinics and adapted the measures to their territory. The CIUSSS are supported by the Montreal Regional Department of Public Health (DRSP) who provided operational support and scientific orientation. The DRSP was also responsible for communicating test results and recommendations to the population.

The main actors who performed the testing were nurses. However, other health care professionals (e.g., doctors, dentists, midwives, physiotherapists, audiologists, etc.) were also called upon to help with testing as the demand increased.

#### Where

Testing took place in designated testing clinics which opened on 2nd April 2020. When the testing volume exceeded the capacities of these clinics, the DRSP deployed mobile clinics to support the CIUSSS.

#### When Implemented

See Figure 1

**FIGURE 1:**
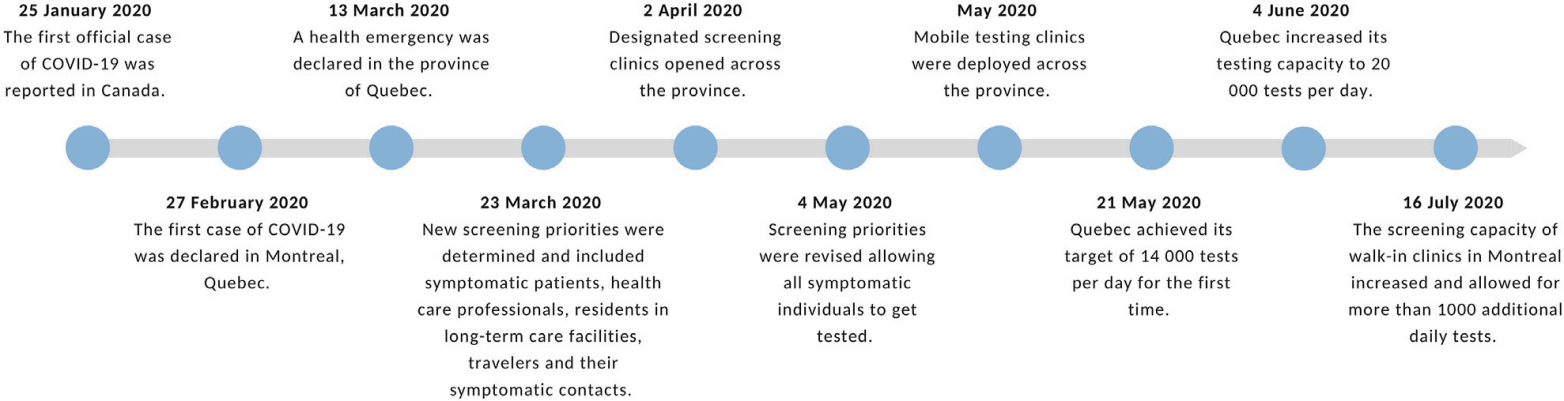
Timeline of important dates related to testing in Montreal and Quebec (Montreal, 2020). Source: Authors’ own work.

#### Variations

Testing capacity significantly increased in May 2020 when Quebec achieved its target to analyse 165 tests per 100,000 people per day and in June 2020 when they could analyse 236 tests per 100,000 people per day. As a result, the criteria to get tested became less restrictive and priorities changed. Due to the high demand for testing, there were also variations regarding the timing in the communication of test results. Most test results were communicated within 24–48 h, although at times it could take up to 7 days. This became more efficient over time during the first wave as the health care system recruited more personnel.

### Bamako, Mali

#### What Materials

Informational materials intended for the public included posters in health facilities on the prevention of transmission and documents describing the testing process. These documents were not available online and could only be found in print formats. These materials were available to the public and professionals in French only.

#### How It Was Planned

Initially, the testing strategy was to test suspect cases and ask contacts to self-isolate. A suspect case was defined as a patient with acute respiratory symptoms. Other criteria for a suspect case included respiratory symptoms with travel to a region with confirmed community transmission, or who had been in contact with a confirmed or suspect case of COVID-19 within 14 days of the onset of symptoms, or whose symptoms had no other obvious cause, or who had been hospitalized. Testing was also required for foreign nationals of countries with high rates of COVID-19 (e.g., China, European countries) as well as for Malians who were repatriated. Subsequently, the decision was taken to systematically test suspect cases, all contacts, outgoing travelers, and ensure that all returning travelers had a negative test dating back to no more than a week prior to landing.

The testing process involved the following steps: 1) when an individual presented COVID-19 symptoms, the health workers at the Reference Health Centers (CSREF) and Community Health Centers (CSCOM) completed a paper form and verbally explained to the individual that they must get tested; 2) an oropharyngeal or nasopharyngeal swab was performed; 3) test results were centralized to the National Institute of Public Health (INSP) laboratory in Bamako; 4) test results were then sent to the technical committee for validation, 5) test results were then sent to the Regional Direction of Health (DRS) and communicated by telephone by personnel from the CSREFS. In the event of a positive result (even if asymptomatic), individuals were sent to a specialized care site located in Bamako and in the regions of Kayes, Tombouctou, Mopti, Ségou and Sikasso.

#### Who Implemented

The national COVID technical management committee put into place by the Ministry of Health and Social Affairs was responsible for coordinating all activities related to testing. The committee consisted of 6 units: coordination, operations, planning, logistics, administration and finances, communication, social mobilization, and community engagement. This committee was also responsible for verifying, validating, and publishing daily COVID-19 testing numbers.

Testing was performed by laboratory technicians, doctors, pharmacists, and nurses and then samples were sent to authorized laboratories for analysis. There were 4 laboratories in Bamako that analyzed all the samples in the country, which were then centralized in the INSP laboratory before being transmitted to the technical management committee.

#### Where

Testing took place in CSREFs and CSCOMs.

#### When Implemented

See Figure 2

**FIGURE 2:**
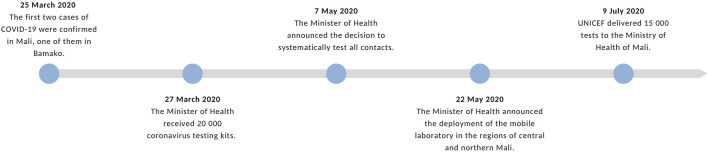
Timeline of important dates related to testing in Bamako and Mali (Bamako, 2020). Source: Authors’ own work.

#### Variations

During the first months of the epidemic, Mali only had about 2,000 tests available, therefore testing was reserved for contacts of cases who were symptomatic. In May 2020, a ministerial decision was then taken to systematically test all contacts regardless of symptoms. In the beginning, only the INSP was responsible for taking samples, however, due to the increase in cases, the rapid intervention teams of the CSREFS and CSCOMS agents were trained to take samples as well. By the beginning of July 2020, there were around 74 tests per 100,000 people that had been performed in Mali in total. There were also variations regarding the communication of results. At the beginning of the first wave, results were communicated approximately 6 h after the test was taken. When the demand for testing increased, results were communicated the next day.

### Paris, France

#### What Materials

Informational materials used for testing included online government resources with a list of all testing sites in the country, public health information, tools to auto-assess symptoms, and instructions to follow for a positive test result [17]. Information for the public was available in a variety of languages. Indeed, the city launched an information campaign at the beginning of the first wave that aimed to reach allophones and more marginalized populations, and videos explaining health measures were made available online in up to 25 different languages [18].

#### How It Was Planned

The national testing strategy and testing priorities evolved in parallel to the evolution of the epidemic and scientific advances in testing. As of 3 March 2020, only people who were identified as possible cases were eligible for testing. At this time, a suspect case was defined as anyone with acute symptoms of respiratory infection who had traveled or stayed in an area with a high risk of exposure (e.g., a country with high community transmission) within 14 days of the beginning symptoms, who had been in close contact with a confirmed COVID-19 case or whose symptoms had no other obvious [19]. As of 11 May 2020, all symptomatic individuals could be tested if they had a medical prescription. As of 25 July 2020, all residents of France regardless of whether they had symptoms could be tested without a medical prescription.

#### Who Implemented

The testing strategy was defined at the national level. Stakeholders involved in the process included France’s President, ministers, the COVID-19 scientific committee, and so forth. Across the French territory, regional health agencies (ARS), prefectures and municipalities were responsible for implementing the testing strategy in their regions. The ARS were also responsible for planning the testing and identifying the facilities where testing could take place as well as the professionals who could perform the tests. In Paris, the Primary Health Insurance Fund (CPAM) managed different health brigades that supported testing. For example, they had brigades responsible for at-home testing. The CPAM was also responsible for contact tracing and would therefore refer contacts of positive cases to get tested.

Healthcare professionals responsible for performing tests included nurses, medical biologists, doctors, as well as medical and nursing students. As demand for testing increased, more professionals became authorized to perform tests with first responders and firefighters assisting during certain moments of need.

#### Where

In Paris, testing took place in laboratories, health centers, and designated COVID-19 testing centers which opened in mid-March 2020. Testing could also be conducted in people’s homes where mobile teams would travel, as well as in mobile clinics set up in different neighborhoods, notably the more disadvantaged neighbourhoods.

#### When implemented

See Figure 3

**FIGURE 3:**
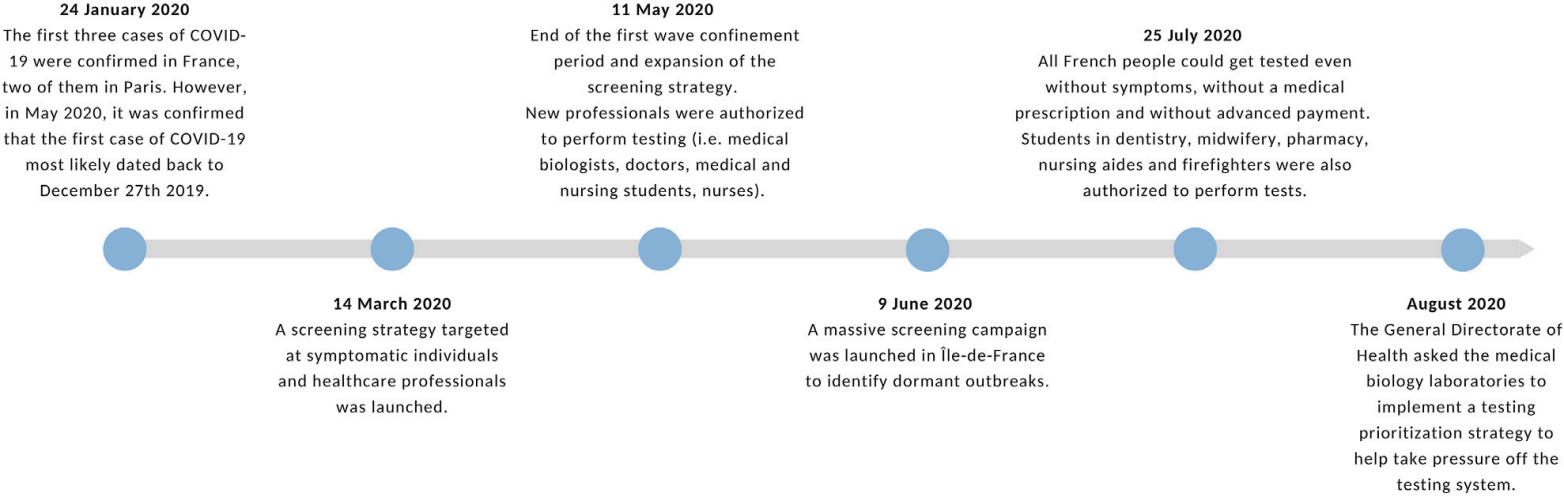
Timeline of important dates related to testing Paris and France (Paris, 2020). Source: Authors’ own work.

#### Variations

In mid-March 2020, France was performing around 11 tests per 100,000 people per day. As they began to reopen, the government had an objective of performing at least 153 tests per 100,000 people per day starting in mid-May 2020. However, by July 2020, only 66 tests per 100,000 people per day were performed in the country [20]. Initially, testing was only conducted in hospitals. The first testing centers in Paris opened in mid-March and were originally serving healthcare professionals and vulnerable populations with a prescription. The testing centers opened gradually to the general population and prescriptions were no longer required [21]. There were also variations regarding the communication of test results. During the summer of 2020, the high demand for testing saturated the system, causing long delays in obtaining a test and the results (test results could take up to 15 days in the Paris region).

### Recife, Brazil

#### What Materials

Informational materials used for mass testing included online public health information and resources to assess symptoms and locate a testing center. This information could be found on the website Pernambuco against COVID-19 (Pernambuco contra a covid-19) and was available only in Portuguese [22]. In March 2020, the state of Pernambuco launched the web application “Atende em casa—Covid 19,” which translates to “at-home consultation.” The application was developed to auto-assess COVID-19 symptoms, make testing appointments, and video chat with a health care professional if needed.

#### How It Was Planned

At the beginning of the first wave, the Central Public Health Laboratory of the State of Pernambuco (LACEN-PE) had a very limited capacity to process tests. Therefore, testing priorities only targeted hospitalized patients with severe flu-like and respiratory virus symptoms. People with mild symptoms were recommended to isolate at home for 14 days and were not tested. In May 2020, new testing priorities included symptomatic health professionals, inmates, public security professionals, household contacts, the elderly, residents of long-term care facilities, and newborn babies whose mothers had tested positive. In June 2020, priorities expanded to include symptomatic essential workers (supermarkets, pharmacies, banks, hospitals, etc.), and patients admitted for surgery. It was only at the end of the first wave, that testing priorities broadened to include asymptomatic contacts.

Testing was recommended for suspect cases, defined as: 1) a symptomatic person with a history of travel in the last 14 days to a country with confirmed COVID-19 transmission or an area with local transmission; 2) a symptomatic person who had been in close contact with a suspected or confirmed case of COVID-19 in the last 14 days; 3) a symptomatic person who had been in contact with a confirmed household case of COVID-19 in the last 14 days. Initially, the LACEN-PE recommended using three swabs to collect samples (two nasopharyngeal swabs and one oropharyngeal swab). However, in May 2020, due to the rising cases and the shortage of testing materials, the LACEN-PE recommended the use of one swab per patient.

#### Who Implemented

The Office of Confrontation to COVID-19 coordinated by the state governor and the State Health Secretariat (SES-PE) was created in March 2020 to analyze the epidemiological situation in the state and deliberate with key stakeholders**.**

The LACEN-PE coordinates the Pernambuco Laboratories network, which is composed of all public and private laboratories that conduct public health-related analyzes. The LACEN-PE was responsible for the daily distribution of COVID-19 testing kits to all state and municipal health units and for the analysis of RT-PCR samples. Health professionals at the Center of Information on Strategic Health Surveillance in Pernambuco (CIEVS-PE) were responsible for immediately notifying suspect and confirmed cases by telephone or email.

#### Where

Testing took place in three testing centers that opened in April 2020. They were exclusively reserved for health professionals. In July 2020, a fourth testing center was opened.

#### When implemented

See Figure 4

**FIGURE 4:**
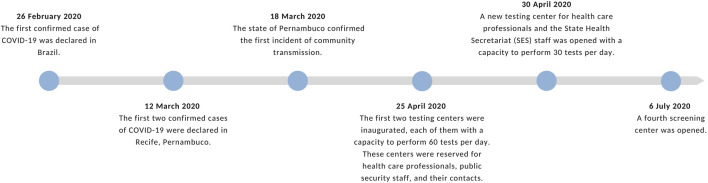
Timeline of important dates related to testing in Recife and Pernambuco (Recife, 2020). Source: Authors’ own work.

#### Variations

The capacity LACEN-PE could only process 10 RT-PCR tests per day in March 2020. After the end of the first wave (September 2020), the LACEN-PE could process over 3,000 tests per day. With this substantial increase in capacity, the eligibility for testing opened. There could be long delays in analyzing and communicating test results, ranging on average from 48 h to 15 days.

## Discussion

Similarities and differences in SARS-CoV-2 testing strategies across the four cities were revealed through in-depth descriptions (see Table 2). The challenges of material and personnel resources during the first wave of the pandemic affected testing capacity and accessibility of the testing across all four sites. Similarly, the testing priorities during the first wave evolved according to the epidemiological situation and the availability of materials, equipment, infrastructure, and human resources in each city. The largest differences in response were related to testing capacity and the timing in terms of implementation and expansion, which can be attributed to the different contexts of each country, including and the state of their health care systems.

**TABLE 2:** Comparative summary of key testing characteristics across the four cities (Montreal, Bamako, Paris, Recife, 2020) [11–22].

	Montreal, Canada	Bamako, Mali	Paris, France	Recife, Brazil
What materials Material Informational	• Material resources used for testing were consistent across cities and included testing kits for RT-PCR tests including swabs for oropharyngeal and nasopharyngeal samples, testing facilities, laboratories, designated refrigerators in laboratories for the storage of samples, transportation materials, analysis kits, and personal protective equipment for personnel • Informational resources were similar across cities and included information on testing sites, auto-assessing symptoms, recommendations to follow, etc. What varied across cities was the availability of this information			
	• Resources were available online or in paper formats at health centers• Resources were available in up to 18 different languages	• Resources were not available online and could only be found in print formats• Resources were only available in French	• Resources were available online or in paper formats at health centers• Resources were available in up to 25 different languages	• Resources were available online or in paper formats at health centers• Resources were only available in Portuguese• The state of Permambuco also had an at-home consultation web application “Atende em casa—Covid 19”
What and how: how it was planned	• In all cities, testing priorities continuously evolved according to the epidemiological situation, testing capacity, and the availability of resources• Testing was initially reserved for symptomatic individuals with flu-like symptoms and travelers• Testing eventually opened to asymptomatic contacts a few months into the pandemic when testing capacity increased			
Who provided the intervention	• The testing strategy was defined at the provincial level by the Ministry of Health and Social Services• The testing strategy was then implemented at the city level by the five Integrated University Health and Social Services Centres and the Montreal Regional Department of Public Health• Testing was performed by nurses and with increasing demand, other health care professionals were called upon to help (i.e., doctors, dentists, midwives, physiotherapists, audiologists, etc.)	• The testing strategy was defined at the national level by the national COVID technical management committee put into place by the Ministry of Health and Social Affairs• Testing was performed by laboratory technicians, doctors, pharmacists, and nurses	• The testing strategy was defined at the national level by the President, government members, the COVID-19 scientific committeeetc.• The testing strategy was then implemented by the Regional Health Agency, prefectures and municipalities• Testing was performed by nurses, medical biologists, doctors, medical and nursing students. As demand for tests increased, first responders and firefighters also assisted with testing	• The testing strategy was defined at the state level by The Office of Confrontation to COVID-19, which was coordinated by the state governor and the State Health Secretariat• The Central Public Health Laboratory of the State of Pernambuco (LACEN-PE) was responsible for the daily distribution of testing kits to state and municipal health units, and for the analysis of samples• Health professionals at the Center of Information on Strategic Health Surveillance in Pernambuco notified suspect and confirmed cases by telephone or email
Where	• Designated COVID-19 testing clinics• Mobile testing clinics	• Reference Health Centers (CSREF)• Community Health Centers (CSCOM)	• Laboratories, health centers, and designated COVID-19 testing centers• Mobile clinics• At-home testing	• Designated COVID-19 testing clinics
Variations	• In all cities, variations in the testing response were mostly due to a shortage of resources (material and human) which directly affected testing capacity, analysis of test results, and communication of results			

During the first wave, main limitations to the testing response across the four cities were due to shortage of RT-PCR tests, laboratory supplies, personal protective equipment, and human resources. This directly affected testing capacity, testing accessibility, the prioritization of tests, confirmed case numbers, the spread of the virus, and lockdown measures [11–14]. Initially, testing was reserved for individuals with acute respiratory and flu-like symptoms as well as travelers coming from areas with high community transmission. It was only a few months into the first wave, that the cities broadened testing priorities to include asymptomatic individuals. Due to this delay, a significant number of cases were most likely undetected, biasing estimates of transmissibility, disease severity, and infection fatality, which are necessary to inform epidemic responses [23, 24]. Another material resource challenge was related to the accessibility of informational material. Testing and public health guidelines were available online and in paper formats in all the cities, except for Bamako, where information was not electronically available. Moreover, in Montreal and Paris, documents were available in many different languages, whereas in Bamako and Recife documents were only available in one language. While the need for information in a variety of languages varies based on the linguistic profiles of countries, studies have shown that reducing language barriers is related to a better understanding of instructions and is also linked to improved health outcomes [25].

While the four cities faced similar barriers related to material and personnel resources, the context of each city and the state of their health care systems prior to the pandemic had a notable effect on overcoming these barriers and mitigating the impact of the pandemic. For example, in 2015, Pernambuco was one of the Brazilian states most affected by the Zika pandemic [26]. From this experience, Recife developed expertise in infectious disease surveillance and managing health emergencies. Conversely, the political context was detrimental to the country’s response as the Brazilian government did not acknowledge the seriousness of the virus and did not follow recommendations from the international scientific community. This resulted in a lack of coordination between federal entities to respond to the pandemic, and states and municipalities did not receive proper financial or material support and lacked human resources [27]. In Mali, the health care system was in a fragile state prior to the COVID-19 pandemic due to the volatile political situation, their economic situation, insufficient funding, and the high prevalence of infectious diseases such as malaria, dengue, and tuberculosis [13, 28]. As a result, Mali was dependent on development aid and had a limited number of testing kits and testing centers which is especially apparent when compared to the other countries. By July 2020 only 74 tests per 100,000 people had been performed in total in all of Mali, which is less than the tests Quebec was performing daily and only a fraction of the tests being performed in France at that same time. This limited testing capacity affected the accuracy of the surveillance and burden estimations [13]. Indeed, there is evidence that low and middle-income countries face more barriers to testing such as a lack of scientific and medical infrastructure, laboratory equipment, testing instruments and testing kits, a lack of trained professionals to perform testing, and difficulties complying with biosafety standards in laboratories [29]. High income countries, such as Canada and France, had the clinical and public health infrastructures necessary to increase testing capacity and accessibility more rapidly, and to develop community-based approaches to testing [30, 31].

### Limitations

COVID-19 epidemiological data including the number of confirmed cases and the number of tests performed is not as readily available for each study city. In Canada, health care and public health are decentralized. Therefore, detailed epidemiological data is available at provincial and municipal levels, and less so at the national level. In contrast, France has a centralized system, and epidemiological data is available at the national level and more difficult to find at the municipal level. Another limitation was that it was difficult to find specific information regarding the testing processes, particularly from the first few weeks of the pandemic as most cities were struggling to adapt to the unprecedented health emergency and were not documenting their response. Moreover, informants were not directly involved in all aspects of the testing process in each of the cities and could not provide the same level of detail. As a result, the individual descriptions are not as equally comprehensive, emphasizing the importance of a systematic method for describing public health interventions from the earliest moment possible.

### Conclusion

This systematic multi-city comparison highlights the main differences and similarities between the SARS-CoV-2 testing response in Montreal, Bamako, Paris, and Recife during the first wave of the pandemic. The use of the TIDieR-PHP framework facilitated comparisons by ensuring that the same key features of the intervention were captured for each city. Intervention descriptions and comparisons across settings have the potential to improve health systems and public health responses. Indeed, successes and failures in other contexts can be learned from and help key stakeholders evaluate how their health systems or interventions are performing [32]. For an appropriate strategic response, it is crucial that interventions be clearly documented to reveal how intervention planning and implementation can be improved and how a particular intervention can be adapted to a different context. Using a framework such as the TIDieR-PHP can improve the quality and comprehensiveness of intervention reporting. In turn, the reporting can be used as a reliable source of information for decision-makers and researchers when planning for future emergencies, implementing an intervention, and building on or replicating findings [8, 9].
